# Effects of bone marrow mononuclear cells on induction of axonal sprouting in cortico-cortical and cortico-striatal pathways in an animal model of cortical ablation

**DOI:** 10.1186/s13104-020-05116-z

**Published:** 2020-06-03

**Authors:** Maria de Fátima dos Santos Sampaio, Arthur Giraldi-Guimarães, Camila da Silva Lourenço, Messias Gonzaga Pereira, Norberto Cysne Coimbra

**Affiliations:** 1 Laboratory of Neuroanatomy and Neuropsychobiology, Department of Pharmacology, Ribeirão Preto Medical School of the University of São Paulo (FMRP-USP), Av Bandeirantes 3900, Ribeirão Preto, São Paulo 14049-900 Brazil; 2 Laboratory of Tissue and Cellular Biology, Centre of Biosciences and Biotechnology of Darcy Ribeiro Northern, Fluminense State University, (UENF), Av. Alberto Lamego, 2000, Campos dos Goytacazes, Rio de Janeiro, 28013-602 Brazil; 3 Laboratory of Clinical and Surgery of Darcy Ribeiro Northern Fluminense State University (UENF), Av. Alberto Lamego, 2000, Campos dos Goytacazes, Rio de Janeiro, 28013-602 Brazil; 4 Laboratory of Plant Breeding of Darcy Ribeiro Northern Fluminense State University, (UENF), Av. Alberto Lamego, 2000, Campos dos Goytacazes, Rio de Janeiro, 28013-602 Brazil

**Keywords:** Bone marrow mononuclear cells, Neocortical plasticity, Cerebral ablation, Cortico-cortical tract, Cortico-striatal tract

## Abstract

**Objectives:**

Many therapies have been proposed in order to investigate the mechanisms of neural repair associated with neurological diseases, including bone marrow mononuclear cells (BMMC) transplantation. However, there is evidence that some encephalic injuries are less responsive to neural repair, such as, for example, cortical ablation. On the other hand, some models of cortical ablation have shown functional recovery after BMMC transplantation. Thus, it is relevant to expand the knowledge of BMMC transplantation-induced neuroplasticity in animal models, considering a promising approach for the rehabilitation of patients with neurological diseases. Using an experimental model of cerebral cortex ablation in adult male Wistar rats, which is known to be poorly responsive to neuroplasticity, the aim of this study was to investigate the effects of BMMC on axonal sprouting in cortico-cortical and cortico-striatal pathways synaptic fields. An anterograde neurotracer was used to evaluate the distribution of axonal fibres.

**Results:**

The results showed that BMMC were not able to significantly induce axonal sprouting in the evaluated synaptic fields. Our results reinforced the idea that cortical ablation may be less responsive to neuroplasticity and the beneficial effects of BMMC therapy depend on the particularities of a neural microenvironment intrinsic to a given cortical lesion.

## Introduction

Injuries to the motor cortex or cortico-spinal tracts interfere with motor control, making movement generation and coordination difficult and causing functional losses [1]. Many therapies have been proposed to investigate the mechanisms of neural repair, including bone marrow mononuclear cells transplantation (BMMC).

BMMC consists of hematopoietic progenitor cells and hematopoietic stem cells, lymphocytes, monocytes and a small number of mesenchymal stem cells (MSCs). They have shown beneficial effects by presenting the ability, among others, to release trophic factors and cytokines that favour neuroprotection and repair of injured tissue orchestrating a sensorimotor functional recovery [2–7].

One of the most important events seen as associated with rehabilitation after central nervous system injury consists in the neuroplasticity. However, it is known that this process is different for each injury [8–13].

In a previous study [14], our group investigated the effects of BMMC on functional recovery after focal cortical ablation in rodents. Given the scientific evidence about neuroplasticity [15–17], we considered the instigating cortical ablation model to evaluate the efficiency of BMMC in the induction of brain plasticity. Thus, this study aimed to investigate the effects of BMMC on induction of axonal sprouting in cortico-cortical and cortico-striatal projections synaptic fields. For this, an anterograde neurotracer was used to evaluate the distribution of axonal fibres in synaptic fields of the contralateral neocortex and ipsilateral neostriatum to the lesion.

## Main text

### Methods

#### Animals and surgery

A total of 30 adult male Wistar rats (weighing 320–400 g), provided by the Darcy Ribeiro Northern Fluminense State University (UENF), with 3–4 months of age were used. All experimental protocols were previously approved by the Commission of Ethics in Animal Experimentation of UENF (process 086/2010). The rats were housed in groups of four per cage with food and water available ad libitum in a temperature-controlled room (22 ± 1 °C) under a 12 h/12 h light/dark cycle (lights on at 7:00 a.m.). The rats were transported to the experimental room in their home cages and left undisturbed for 1 h prior to the experiments.

Telencephalic ablation was performed by cerebral cortex aspiration, as previously described [8]. Briefly, after anaesthesia with ketamine hydrochloride at 90 mg/kg, i.p. (Ketamine Agener, União Química Farmacêutica Nacional, Embu-Guaçu, SP, Brazil; 0.2 mL of 10% solution) and xylazine hydrochloride at 10 mg/kg, i.p. (Dopaser, Hertape/Calier, Juatuba, Minas Gerais, Brazil), the left frontoparietal cortex (+2 to −6 mm A.P. from the bregma) was exposed and aspirated with a pipette tip (1 mL) attached to a vacuum pump. A piece of collagen haemostatic sponge was put inside the lesion, the skin was sutured, and the animals were kept warm under a hot lamp and returned to colony room after recovery from anaesthesia.

#### BMMC obtaining and transplantation

Bone marrows were obtained from donor naïve rats (n = 3), as previously described [2]. The animals were euthanised with a lethal dose of ketamine hydrochloride (i.p.) and xylazine hydrochloride (i.p.). BMMCs were collected and washed with phosphate-buffered saline (PBS). Following cell count, they were re-suspended in sterile physiological saline, and the final concentration was approximately 3 × 10^7^ BMMCs/0.5 mL.

Twenty-four hours after the surgical procedure, the animals were anaesthetised with ketamine hydrochloride (90 mg/kg, i.p.) and xylazine hydrochloride (10 mg/kg, i.p.) and treated with BMMC or vehicle (PBS) injected through the left jugular vein. The skin was sutured, and the animals were kept warm under a hot lamp and returned to colony room after recovery from anaesthesia.

#### Experimental design

The animals were randomly divided into experimental groups. NAÏVE (animals without lesion), CONT (animals submitted to cortical ablation and treated with vehicle), BMMC (animals submitted to cortical ablation and treated with mononuclear cells of bone marrow). The therapeutic window was chosen based on our previous studies [5].

Animals from the NAÏVE (n = 7), CONT (n = 6) and BMMC (n = 9) groups were subjected to microinjections of an anterograde neural tract tracer 60 days after the surgical procedure. In all groups, the animals were euthanized 8 days after the neural tract tracer microinjections.

#### Characterisation of the lesion

For the characterisation of the lesion, four untreated animals were euthanised 72 h after cortical ablation with a lethal dose of ketamine hydrochloride (i.p.) and xylazine hydrochloride (i.p.). The brain was rapidly removed, according Sampaio et al. [5]. The slices were immersed for 30 min into 2% 2,3,5-triphenyltetrazolium chloride (TTC, T8877/1 Sigma-Aldrich) solution at 37 °C. Digital images were captured from reacted slices with a camera coupled to a dissecting microscope and to a computer.

#### In vivo neural tract tracing procedure

Sixty days after cortical ablation, the animals were anaesthetised with xylazine hydrochloride (10 mg/kg, i.p.) and ketamine hydrochloride (90 mg/kg, i.p.), and fixed in a stereotaxic apparatus. A small trepanation was performed in the calvaria over the sensorimotor cortex in the hemisphere contralateral to the lesion. Using a stereotaxic coordinates obtained from a rat brain atlas (anteroposterior axis, +1.2 mm, mid-lateral axis, − 2.7 mm), the 10% Alexa Fluor 546-conjugated dextran neural track tracer solution (Dextran, Alexa Fluor 546; 10,000 MW, Anionic Fixable, Life Technologies, Austin, TX, USA) was microinjected into the primary motor cortex (M1) through a Hamilton syringe. The injections were made in the depths of 1.8 mm and 2.0 mm from Bregma, with a total volume of solution injected of 0.5 μL.

#### Histological analysis

After 8 days of the neural tract tracer injection, each animal was euthanised with a lethal dose of with ketamine hydrochloride (i.p.) and xylazine hydrochloride (i.p.). Immediately after, the encephalon was perfused through the left ventricle with physiological saline solution (NaCl, 0.9%), followed by 4% paraformaldehyde solution in phosphate buffer (0.2 M, pH 7.4). The encephalon was rapidly removed and incubated in 20% sucrose dissolved in PBS (0.2 M, pH 7.4) for 24 h, soaked in the Tissue-Tek O.C.T. compound and immersed in liquid nitrogen for freezing. It was then sectioned in the coronal plane (30 μm thick), at − 20° C in a cryostat (CM 1950 Leica, CM 1950 Leica, Wetzlar, Germany) and the slices arranged in gelatinised slides, which were subsequently visualised under a motorised fluorescence microscope (AxioImager Z1 with APOTOME II; Zeiss, Oberkochen, Germany).

#### Data analyses

The images were selected and treated using the ImageJ software (NIH). From the efficacy of the neural tract tracing, in each two brain sections slides per animal, 3 fields were analysed as follow: Neocortex surrounding the lesioned area and the neostriatum ipsilateral and contralateral to the cortical brain lesion (Fig. 2j). The mean per animal was calculated in respective areas and then the mean for each experimental group was plotted for the statistical analyses. The percentage of axonal fibres was computed based on the number of axons identified per unit of visual field area.

Data from the experiment were submitted to the one-way ANOVA, followed by Tukey’s post hoc test. Values were reported as the mean and standard error of the mean (S.E.M.); p-values < 0.05 were considered statistically significant. The distribution of the variables passed the normality test (Shapiro–Wilk normality test).

### Results

#### Histochemical characterisation of the cortical lesion

**Fig. 1:**
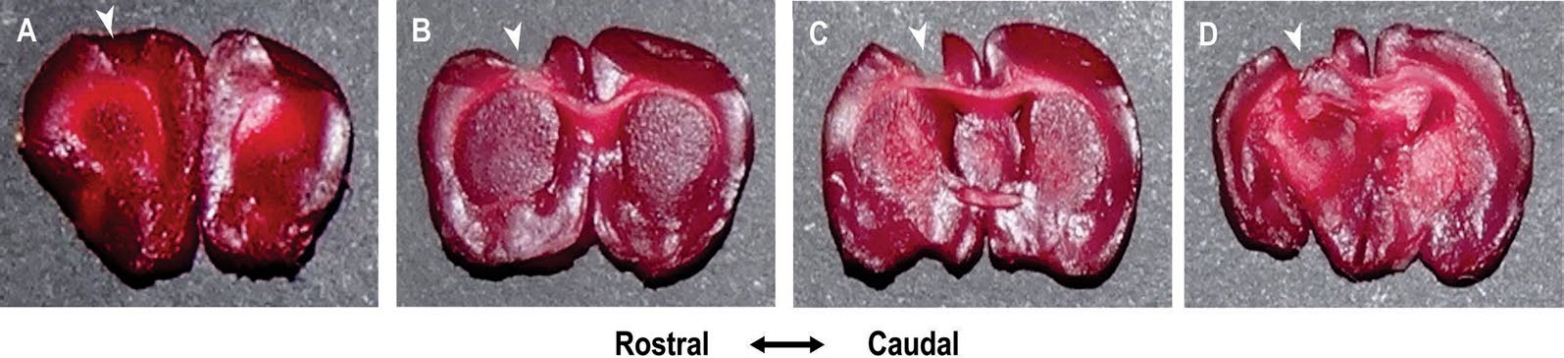
Extension of the cortical ablation procedures. The figure shows sequential coronal sections (2 mm) of Wistar rats brain 72 h after each cortical lesion, submitted to the histochemical reaction with 2,3,5-triphenyltetrazolium chloride (TTC), which reddens the viable tissue. **a**–**d** Histological sections of a brain reacted after ablation, observing the removal of the sensorimotor area (white arrowheads at the top of each slice)

Using TTC, it was demonstrated that all lesioned animals, 72 h after each the surgical procedure, showed in the sensory-motor region, loss of neocortical tissue restricted to the sensory-motor cortical area when submitted to the cortical ablation, as shown in Fig. 1.

#### Quantitative analysis of cortico-cortical and cortico-striatal projections of naïve rats and those submitted to a unilateral cortical ablation after BMMC or vehicle transplantation

Statistical analysis was performed exclusively with data from the animals that presented evidence that microinjection in the primary motor cortex was successfully performed.

**Fig. 2:**
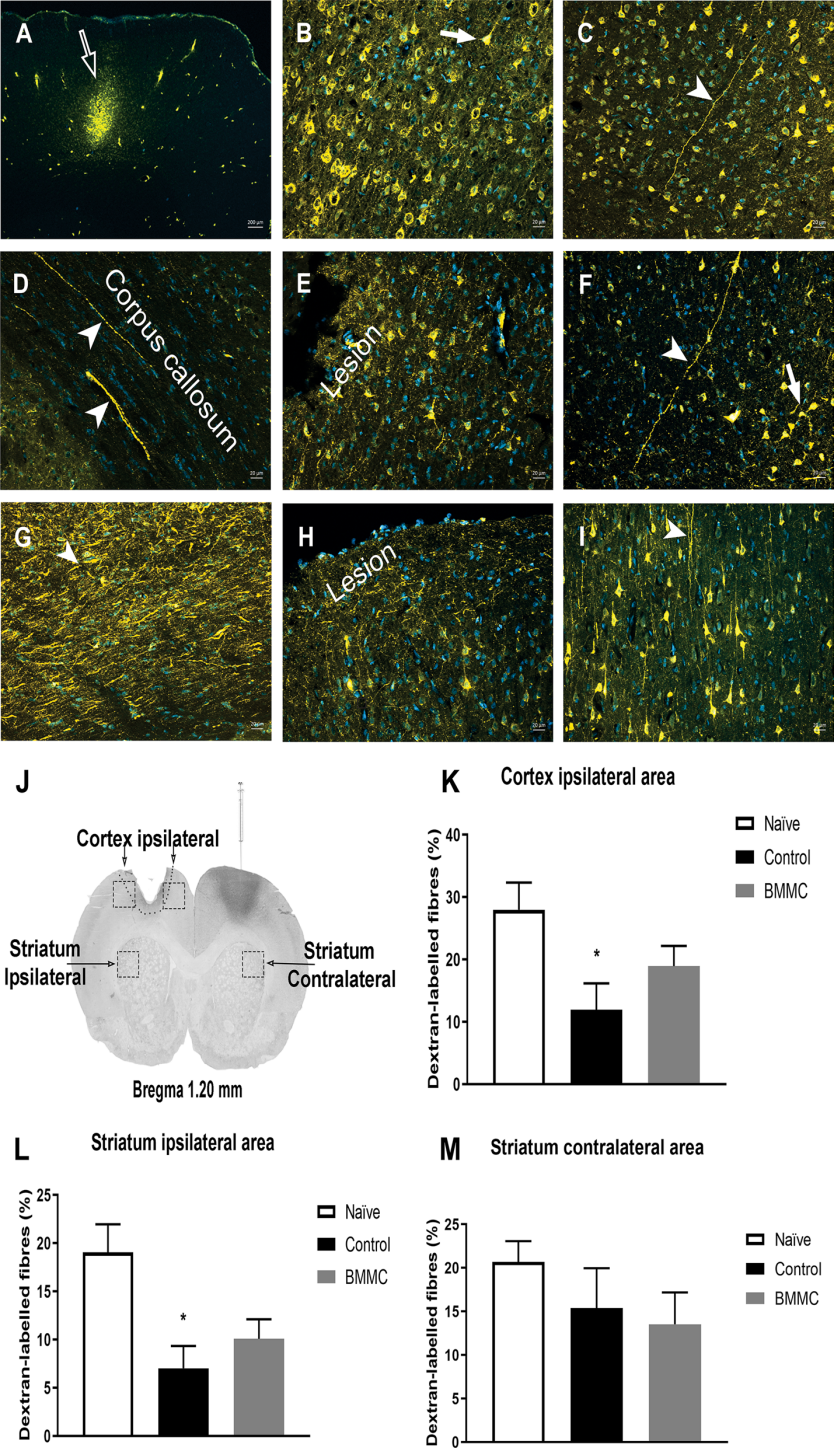
Photomicrographs of transverse sections of “NAÏVE” Wistar rats brain (**a**–**c**), of control (CONT) Wistar rats brain (**d**–**f**), and BMMC-treated (BMMC) Wistar rats brain (**g**–**i**). **a** Representative site of microinjection of the anterograde Alexa Fluor 546-conjugated dextran neural tract tracer in the primary motor cortex (white open arrow). **b** Neurons (white arrow) situated in the cerebral cortex external pyramidal layer ipsilateral to the site of cortical deposits of the neurotracer that send projections (white arrowheads in **c** and **d**) to the contralateral neocortex. **d** Transverse section of the corpus callosum showing (white arrowheads) axons of cortico-cortical (**e** and **f**) and cortico-neostriatal (**g**) connexions. **h** Cerebral cortex ipsilateral to lesion showing neurotracer-labelled perikarya and fibres in the internal pyramidal layer (**h**) and in the fusiform cortical layer (**i**). **j** A histologically confirmed representative site of injection of the anterograde neural tract tracer Alexa Fluor 546-conjugated dextran in the primary motor cortex, on the right, illustrating (squares) the quantified areas for analysis of cortico-cortical and cortico-striatal projections. The effect of the transplantation of BMMC or vehicle-treatment on axonal sprouting in cortical layers surrounding the brain lesions (**k**) and in the neostriatum ipsilateral (**l**) and contralateral (**m**) to the cortical ablation were also demonstrated. Data were represented as mean ± S.E.M.; n = 6-9 rats per group; *p < 0.05, in comparison to the NAÏVE group, according to a one-way ANOVA followed by Tukey’s post hoc test. Scale bars: 200 μm on the panel **a**; 20 μm on the panels **b**–**i**

Results of quantitative morphological analysis showed that intravenous treatment with BMMC did not significantly increase the number of neurotracer-labelled axonal fibres in areas of the neocortex and neostriatum ipsilateral to the lesion and in the neostriatum contralateral to the cortical ablation (Fig. 2a–i). Analysis of the neural tissue ipsilateral to the cortical lesion and surrounding the brain damage, according to the one-way ANOVA, revealed a significant effect of the treatment (F_2,16_ = 3.992, p < 0.05). The neocortical lesion with cortical tissue ablation of animals not treated with BMMC (CONT group) was followed by a significant decrease in the number of neurotracer-labelled axonal fibres in the cortical layers surrounding brain injury compared to healthy rodents (NAÏVE group) (Tukey’s post hoc test; p < 0.05). These findings are shown in Fig. 2k.

Regarding the neostriatum ipsilateral to the brain lesion, there was a significant effect of the treatment (F_2,13_ = 6.444; p < 0.05), according to one-way ANOVA. Control injured animals (CONT) showed a reduction in neurotracer-labelled axonal fibres when compared to the healthy group (NAÏVE group). These data are shown in Fig. 2l. With respect to the caudate-putamen contralateral to the lesion, according to one-way ANOVA, there was any significant difference between the experimental groups (F_2,17_ = 1.143; p > 0.05), as shown in Fig. 2m.

Interestingly, the significant decrease of neurotracer-labelled fibres in lesioned animals was reversed by BMMC treatment, as shown in Fig. 2k and l.

## Discussion

Our findings showed that BMMC treatment did not induce an increase in axonal fibres in areas of the contralateral cortex and ipsilateral and contralateral neostriatum to the lesion of animals injured by cortical ablation. There are few studies in Literature regarding cell therapy in experimental models of cortical ablation, possibly because it represents a less frequent clinical situation when compared to stroke or head trauma. Cortical ablation resembles a condition of surgical removal of a brain tumour [14].

Regarding neuroplasticity after injury, there is evidence that cortical ablation has been shown to be poorly responsive for structural neuroplasticity [9–11]. Therefore, it became an attractive model for this work, which aimed to extend the studies of BMMC efficiency, now addressing the induction of neuronal circuit remodelling, in an attempt to better elucidate the relationship of neuroplasticity and motor functional recovery.

The results of this study corroborate previous findings in Literature [11, 18] showing that after cortical ablation in a sensorimotor area there was little responsiveness to cortico-cortical and cortico-striatal neuroplasticity. In this study, we also observed that treatment with BMMC was not able to significantly reverse the effect of the cortical lesion.

Carmichael and Chesselet [19] contributed to that issue, showing that different sensorimotor cortex lesions may promote different responses in the induction of axonal sprouting, even considering injuries of the same location and extension. These authors, using experimental models of ablation and ischemia, showed that there was an increase in axonal labelling in the medial cortex and striatum, both ipsilaterally to the lesion, but only in animals submitted to ischemia. Thus, attention was paid to a brain reorganisation dependent on the type of injury induced in rats, reinforcing the previous results by Szele et al. [8], who indicated that lesions induced by aspiration promoted a greater inhibition of structural plasticity, unlike cortical ischemic lesions.

According to Freitas et al. [13], one of the possible causes of not observing a significant response to structural plasticity after cortical ablation is due to the injury neuropathological mechanism itself. Probably pathologies such as stroke are more responsive to this event, as it speeds up substantial necrosis and favours a significant inflammatory response [11, 18, 20, 21].

With regard to therapy, the results pointed out by Michael Chop reinforce that cell therapy with bone marrow stromal cells (MSCs) is a promising strategy in the induction of neuroplasticity and in functional sensorimotor recovery after ischemia [22, 23]. However, the experimental model and treatment differ from those used in this work.

Research has shown that BMMC has the capacity for neuroprotection and neural repair, but it is essential to seek therapeutic strategies that interact positively with the biological processes inherent to each pathological process.

## Limitation

The limitation of the present work is showing only results regarding the effect of the treatment with BMMC on the induction of axonal sprouting, in a cerebral cortical ablation model, without comparisons with an ischemic group. However, it was considered that the cortical ablation model is described as not very responsive to plasticity, whereas, in ischemic models, treatment with cells derived from bone marrow promoted significant results.

## Data Availability

Data supporting our finding are available by the corresponding author.

## Abbreviations

ANOVA: Analysis of variance
BMMC: Bone marrow mononuclear cells
CEUA: Commission of Ethics in Animal Experimentation
M1: Primary motor cortex
MSCs: Mesenchymal stem cells
PBS: Phosphate buffered saline
TTC: 2,3,5-Triphenyltetrazolium chloride
